# Evaluation and Analysis of Costs Associated with Prophylaxis of Recurrent Urinary Tract Infections (RUTIs) in Women

**DOI:** 10.3390/microorganisms13020393

**Published:** 2025-02-11

**Authors:** José Emilio Hernández-Sánchez, Julius Jan Szczesnieski, Bárbara-Yolanda Padilla-Fernández, Carmen González-Enguita, Javier Flores-Fraile, María Fernanda Lorenzo-Gómez

**Affiliations:** 1 Department of Surgery, University of Salamanca, 37008 Salamanca, Spain; jemilio.hernandez@fjd.es (J.E.H.-S.); padillaf83@hotmail.com (B.-Y.P.-F.); mflorenzogo@yahoo.es (M.F.L.-G.); 2 Urology Department, General Hospital of Villalba, 28040 Madrid, Spain; cgenguita@fjd.es; 3 Department of Urology, University Hospital of Getafe, 28905 Getafe, Spain; jszczesniewski@usal.es; 4 Multidisciplinary Renal Research Group of the Institute for Biomedical Research of Salamanca (IBSAL), 37007 Salamanca, Spain

**Keywords:** recurrent urinary tract infections, costs urinary tract infections, prophylaxis urinary tract infections

## Abstract

To determine the variations in the costs of the prophylaxis of recurrent urinary tract infections (RUTIs) among different prevention protocols, a prospective observational multicenter study on 1614 women receiving RUTI prophylaxis was conducted. The patient groups were as follows: Group A (n = 444): conventional suppressive antibiotic therapy; Group V (n = 732): polyvalent bacterial vaccine; and Group O (n = 438): other adjuvant measures. The variables were age, body mass index, American Society of Anesthesiologists (ASA) physical status classification scale, cost of prophylaxis, duration of the RUTI, number of visits for primary and specialized care, number of UTIs, cost of urinalysis, urine culture, urine cytology, and days of sick leave. The mean age was 57.71 years but was found to be lower in GV. The mean expenditure on UTI prophylaxis and treatment per patient was EUR 4908.07, but this found to be higher in GO. Emergency primary care visits were more frequent in GA. The ordinary scheduled visits to primary care visits were more frequent in GV and GO. The mean successive visits was 2.47 and was shown to be lower in GV. The mean expenditure on successive visits was EUR 341.64 but was found to be lower in GV. The mean number of UTIs was 4.83 at 3 months after finishing prophylaxis and 5.01 at 12 months, and it was observed to be lower in GV. Less frequent VCU usage, older age, more ASA III, more frequent use of urinalyses, urine cultures, ultrasounds, and CT scans were associated with higher costs. In GO, IVU was associated with higher costs. The total expenditure related to RUTIs is associated with older age and the number of RUTIs, a poorer general health status, and the use of urinary tract ultrasounds and CT scans. The use of VCUs instead of ultrasounds and CT scans is cost-effective in the management of RUTIs in older women. Immunoprophylaxis is more cost-effective in reducing the number of visits to the primary care emergency room, the number of successive visits to the Urology Department, the number of intercurrent infections, and the need for urinalyses, urine cultures, CT scans, and ultrasounds in the primary care emergency room.

## 1. Introduction

Urinary tract infections (UTIs) are defined as the presence of a significant number of bacteria in the urinary tract that leads to an inflammatory response of the urothelium [1]. The presence of bacteria in the urinary tract may or may not cause symptoms [2]. Both isolated UTI episodes and RUTIs negatively affect patients’ quality of life [3,4].

Recurrent urinary tract infections (RUTIs) affect 5–10% of women globally, representing a significant burden on healthcare systems and society. These infections negatively impact patients’ quality of life and often lead to substantial direct and indirect costs. RUTIs are defined as two or more UTI episodes within six months or three or more episodes within one year [3]. Their high prevalence, coupled with the growing concern of antimicrobial resistance due to frequent antibiotic use, underscores the need for effective and sustainable prophylactic strategies [3]. The European Urology Association, in its guidelines, suggests using non-antibiotic measures first and recommends antibiotic prophylaxis only when those have failed [3].

Among the innovative approaches to RUTI prevention, Uromune^®^, a sublingual polybacterial vaccine, has emerged as a promising option [5,6]. This vaccine stimulates the immune system by exposing it to inactivated bacterial antigens derived from common uropathogens. Recent studies have demonstrated its ability to reduce healthcare resource utilization and the associated costs, highlighting its potential as an effective prophylactic measure for RUTIs [7,8].

Economic evaluations play a crucial role in comparing healthcare interventions, particularly in conditions like RUTIs, where costs extend beyond direct medical expenses to include productivity losses and intangible burdens [4,6,9]. Cost-effectiveness analyses provide valuable insights into balancing clinical outcomes and resource use, offering a framework for prioritizing interventions that deliver the greatest health benefits [6,9].

This study aims to evaluate and compare the costs associated with different prophylaxis protocols for RUTIs and to identify the key factors influencing these cost differences. By doing so, it seeks to inform clinical decision-making and contribute to the development of more cost-effective and patient-centered approaches to RUTI management.

## 2. Methods

This prospective, observational, multicenter study included 1614 women diagnosed with RUTIs who initiated antibiotic or non-antibiotic prophylaxis. Eligible participants were aged 18 years or older. The study began in February 2019.

### 2.1. Patient Selection

General practitioners affiliated with the Renal Urological Multidisciplinary Research Group (GRUMUR) at the Institute of Biomedical Research of Salamanca (IBSAL, Salamanca, Spain) identified potential participants during routine consultations. Patients were informed about the study, provided with an information sheet, and asked to sign an informed consent form. After consent was obtained, the required data forms were completed. Subsequently, all participants underwent an evaluation by the Urology Department at one of the participating hospitals: Virgen de la Vega University Hospital of Salamanca, Clinical University Hospital of Salamanca, Virgen del Castañar Hospital of Béjar, or Nuestra Señora de Sonsoles University Hospital of Ávila.

The inclusion criteria were as follows: Women aged 18 years or older who were under the care of participating physicians; met the RUTI prophylaxis prescription criteria; and signed the informed consent form. The exclusion criteria included urinary lithiasis, moderate-to-severe urinary incontinence, generalized immunosuppression, or the inability to provide informed consent.

### 2.2. Study Protocol

At the initial visit, all participants underwent a standardized assessment that included anamnesis, physical examination, urinalysis, and urine culture. Additional diagnostic tests, such as urinary tract ultrasound, voiding cystourethrography (VCU), intravenous urography (IVU), or computed tomography (CT), were performed as needed in order to adhere to the exclusion criteria.

The prescribing physicians had the discretion to select the most appropriate treatment protocol for each patient, provided they met the inclusion criteria and had no exclusion factors. Patients attended scheduled follow-up visits at 3, 6, and 12 months, with annual follow-ups thereafter. Patients who did not complete the follow-up visits were not included in the analysis.

### 2.3. Study Groups

Group A (GA: n = 444) was subjected to conventional suppressive antibiotic prophylaxis, administered nightly for six months. Subgroups included the following:

- GAa: ciprofloxacin: 500 mg of oral ciprofloxacin every night for 6 months;
- GAb: fosfomycin: 500 mg of oral fosfomycin every night for 6 months;
- GAc: cotrimoxazole: oral trimethoprim 160 mg/sulfamethoxazole 800 mg every night for 6 months;
- GAd: nitrofurantoin: 50 mg of oral nitrofurantoin for 6 months;
- GAe: amoxicillin with or without clavulanic acid: 500 mg of oral amoxicillin every night for 6 months.

Group V (GV: n = 732) received prophylaxis using a sublingual bacterial preparation of whole-cell inactivated bacteria derived from a urine sample (Uromune^®^. Inmunotek©. Madrid, Spain. Q-Pharma, Alicante, Spain), administered as two sublingual puffs of the vaccine once a day for three months [6].

Group O (GO: n = 438) was treated with non-antibiotic and non-polyvalent-bacterial-vaccine adjuvant measures. Subgroups included the following:

- GOa: pelvic floor biofeedback (BFB): 20 min sessions of pelvic floor BFB with surface electrodes [10], controlled by the Rehabilitation Departments of the participating hospitals;
- GOb: oral D-mannose (Manosar^®^): 1 portion stick every 24 h for 6 months;
- GOc: bladder glycosaminoglycan instillations (Cystistat^®^: 50 mL with 40 mg of sodium hyaluronate): 1 instillation per week for 4 weeks, followed by 1 instillation every 2 weeks for 2 months, and 1 instillation per month for 4 months;
- GOd: topical vaginal estrogens (Colpotrofin^®^ vaginal cream 10 mg/g^®^): one dose every night for 6 weeks.

### 2.4. Study Variables

The data collected were categorized as follows:

- General data: Age, body mass index (BMI), general health status (measured using the American Society of Anesthesiologists [ASA] physical status classification scale), comorbidities, concurrent treatments, history of substance use, and past surgeries.
- Specific data: Duration of RUTI clinical evolution, number of days receiving prophylaxis, number of UTI episodes, frequency of emergency and follow-up visits to primary or specialized care, days of sick leave, and use of diagnostic imaging tests (e.g., ultrasound, VCU, IVU, CT), urinalysis, urine culture, and urine cytology.
- Economic data: Costs per prophylaxis unit, total prophylaxis course, imaging tests, laboratory tests, and additional medications (e.g., NSAIDs, gastric protection drugs).

The reference costs for treatments and prophylaxis were sourced from the official rates established by the Regional Healthcare Service (Gerencia Regional de Salud de Castilla y León [Sacyl], Spain).

### 2.5. Statistical Analysis

The results were analyzed using statistical tools to ensure meaningful interpretations. Descriptive statistics summarized the data, providing measures such as mean, median, standard deviation, and range for continuous variables like age and BMI.

The comparative analyses included the following:

- Student’s *t*-test: For the comparison of two groups with normally distributed data.
- Chi-squared test: To assess differences in categorical variables.
- ANOVA: For comparing means across multiple groups with normal data; Scheffe’s method was used for post hoc analyses.

Univariate and multivariate regression analyses explored the associations and controlled for confounders, examining factors influencing expenditure, such as age, ASA status, and RUTI episodes.

Analyses were conducted with the NSSS2006/GESS2007 software (Version 2006/2007) [11]. Statistical significance was set at *p* < 0.05, and post hoc tests were applied to identify specific group differences.

### 2.6. Ethical Considerations

The 2020/230/14 study protocol was approved by the Ávila Drug Research Ethics Committee (CEIM) (Ávila 05004, Spain). All authors declared no competing interests.

### 2.7. Funding Source

The study was supported by the Renal Urological Multidisciplinary Research Group (GRUMUR) of the Institute of Biomedical Research of Salamanca (IBSAL), 37007 Salamanca, Spain.

## 3. Results

A total of 1614 women were included in the study, with a mean age of 57.71 years. The mean age was significantly lower in GV (*p* = 0.00057). In GO, the mean age was 61.11 years, with subgroup GOa presenting the lowest mean age (*p* = 0.000001). Detailed data for each subgroup are presented in Supplementary Table S1.

The mean BMI across the cohort was 27.88 kg/m^2^. BMI was significantly lower in GV compared to the others (*p* = 0.00028). The analysis of comorbidities showed higher rates of hypertension, type 2 diabetes mellitus, dyslipidemia, and obesity in GA compared to GV (all *p*-values < 0.001). Similar trends were observed when comparing GA to GO, where additional conditions, including hiatus hernias, depression, and insomnia, were also significantly more prevalent (*p*-values ranging from 0.0001 to 0.0195) Table 1.

Hysterectomies were significantly more common in GA compared to both GV (*p* = 0.0001) and GO (*p* = 0.0008). Eutocic deliveries were also more frequent in GA compared to GV (*p* = 0.0132). Additionally, patients in GA exhibited a higher prevalence of NSAID allergies compared to GV (*p* = 0.0110) and GO (*p* = 0.0002). No significant differences were observed in the prevalence of active smoking between the groups (*p* = 0.6825), although GV had a significantly higher proportion of ex-smokers compared to GO (*p* = 0.0336).

The mean duration of clinical evolution of RUTIs was 5.02 years, significantly shorter in GV (*p* = 0.0001). In GO, the mean duration was 2754.80 days, with no significant differences among its subgroups (*p* = 0.0639).

At the beginning of the study, the urine cultures revealed notable differences in the microbial profiles among the groups. *Enterococcus faecalis* was more frequently detected in GV (18.03%) compared to GA (12.84%) (*p* = 0.0217). Conversely, *Escherichia coli* was significantly more prevalent in GA (77.70%) than in GV (61.89%) (*p* = 0.0001) and GO (68.49%) (*p* = 0.0023). *Candida glabrata* was isolated exclusively in six patients (1.37%) in GO. *Pseudomonas aeruginosa* was more frequently found in GA (1.35%) and GV (0.82%) compared to GO (0%).

Urine cytology at the beginning of the study revealed that leukocyturia was more frequent in GV (4.33%) than in GA (2%) (*p* = 0.0344), while squamous cells were more frequently observed in GA (1.33%) than in GO (0%) (*p* = 0.0148).

### 3.1. Number of Days of Prophylaxis Administration

The mean duration of prophylaxis administration was 132.37 days, with the shortest duration observed in GV (*p* = 0.00001). In GO, the mean duration was 67.28 days, with subgroup GOc showing the shortest duration (*p* = 0.00001).

### 3.2. Expenditure in UTI Prophylaxis and Treatment (Euros)

The mean cost per dose of prophylaxis was EUR 108.76, significantly lower in GA compared to the other groups (*p* = 0.00001). The standard GA instillation protocol cost was EUR 2384.88, including the materials and nursing activities.

Table 2 highlights the significant differences in the number of days of prophylaxis, associated costs, and healthcare resource utilization.

- Number of Days of Prophylaxis: Group A (GA) consistently maintained a 183-day regimen. GV had a shorter duration (90 days), and GO demonstrated the greatest variability, especially in subgroups GOa (39.03 days) and GOc (23.05 days).
- Expenditure per Dose of Prophylaxis: Ciprofloxacin (GAa) had the lowest per-dose cost (EUR 2.31), while amoxicillin/clavulanic acid (GAe) was the most expensive (EUR 8.73). Within GO, bladder glycosaminoglycan instillations (GOc) showed the highest costs (EUR 851.60), followed by vaginal estrogens (GOd, EUR421.50).
- Number of Successive Visits: GV required the fewest successive visits (mean = 1.09), compared to GA (mean = 5.09) and GO (mean = 2.12). Subgroup-level analysis revealed lower visit rates in GOb (oral D-mannose, mean = 2.31) and GOa (pelvic floor biofeedback, mean = 3.14).
- Expenditure During Successive Visits: Successive visit costs were significantly lower in GV (EUR 151.37, *p* < 0.0001) compared to GA (EUR 703.62). Within GO, glycosaminoglycan instillations (GOc) were the most expensive (EUR 449.35).

**Table 2:** Summary of Prophylaxis Duration, Costs, and Healthcare Utilization by Group. ANOVA test was employed to perform the analysis; Scheffe’s method was used for post hoc analyses.

	Number of Days of Prophylaxis		Expenditure per Dose of Prophylaxis (EUR)			Expenditure in First Visits (EUR)		Number of Successive Visits in Urology			Expenditure in Successive Visits (EUR)		
	Mean	Sd	Mean	Sd	Mean	Sd	*p*	Mean	Sd	*p*	Mean	Sd	*p*
GA	183	0.5	6.80	3.21	81.72	15.07	0.0001	5.09	2.74	0.0001	703.62	379.10	0.0001
GAa			2.31	0.5				3.45	2.09		476.69	288.81	
GAb			4.64	0.13				3.26	2.13		450.57	294.42	
GAc			3.12	0.2				3.56	2.29		492.37	317.23	
GAd			3	0.10				3.41	2.48		471.83	343.06	
GAe			8.73	2.73				3.15	1.94		435.19	267.75	
GV	90	0.10	180	0.10	86.60	12.27		1.09	0.39		151.37	54.04	
GO	151.88	76.90	587.71	294.15	85.88	12.95		2.12	1.73		292.67	239.33	
GOa	39.03	52.97	310.60	0.10				3.14	2.38		433.68	329.16	
GOb	188.39	14.99	234.36	0.30				3.21	1.79		443.24	248	
GOc	23.05	51.83	851.60	95.49				3.25	1.84		449.35	254.82	
GOd	102.14	91.96	421.5	0.50				3.71	2.72		512.53	375.73	

These data emphasize the cost-saving potential of GV, particularly in reducing successive visits and associated expenditures. In contrast, GA displayed significant variability, with ciprofloxacin emerging as the most economical antibiotic option.

### 3.3. Number of Visits and Expenditures

First visits for self-treatment or primary care emergencies were more frequent in GA compared to GV (*p* = 0.0019 and *p* = 0.0001, respectively) and GO (*p* = 0.0001 and *p* = 0.0029, respectively). Conversely, GV and GO surpassed GA in the number of scheduled primary care visits (*p* = 0.0001). Specialized care emergency visits were more frequent in GO compared to GV (*p* = 0.0103).

The mean number of successive visits was 2.47, significantly lower in GV (*p* = 0.0001). The mean expenditure for successive visits was also lower in GV (EUR 341.64; *p* = 0.0001), compared to GA and GO.

### 3.4. Expenditure in Imaging Tests (Euros)

The mean expenditure on imaging tests was EUR 326.37, with no significant differences observed between groups (*p* = 0.1017). The mean number of UTIs in the three months following prophylaxis was 4.83, significantly lower in GV (*p* = 0.0001). After one year, the mean number of UTIs was also lower in GV (5.01; *p* = 0.0001).

### 3.5. Number of UTIs After Ending Prophylaxis and Days of Sick Leave

**Figure 1:**
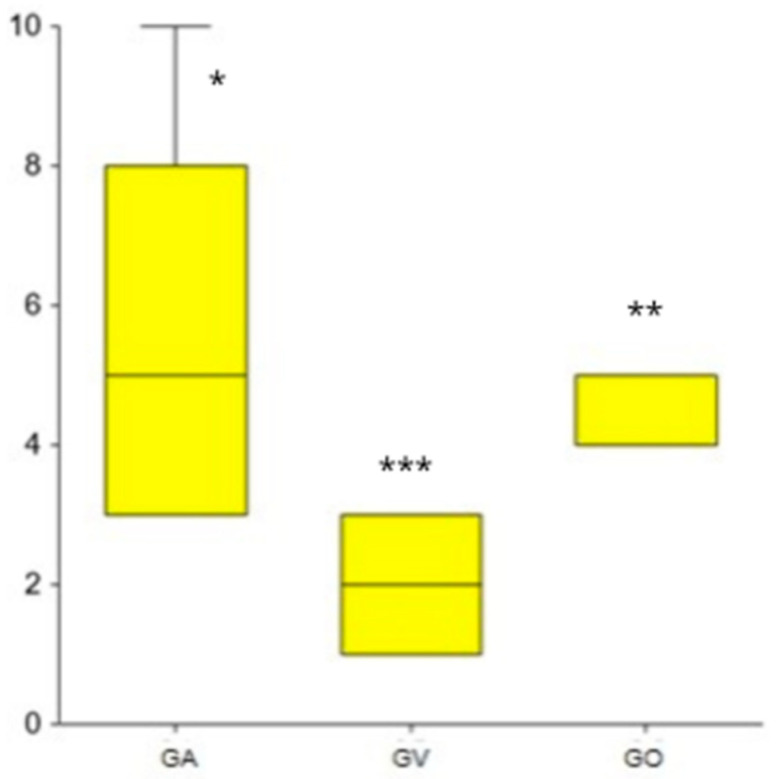
Number of UTIs one year after finishing the prophylaxis in GA, GV, and GO. * GA, one outlier case, ** GO: two outliers cases, *** GV: three outliers cases.

The mean number of urinary tract infections (UTIs) within three months after completing prophylaxis was 4.83, significantly lower in GV (*p* = 0.0001). Similarly, the mean number of UTIs one year post-prophylaxis was 5.01, also significantly lower in GV (*p* = 0.0001) (Figure 1).

The mean duration of sick leave was 5.85 days, with no statistically significant differences observed between the groups (*p* = 0.20970).

### 3.6. Multivariate Analysis: Multiple Regression

**Figure 2:**
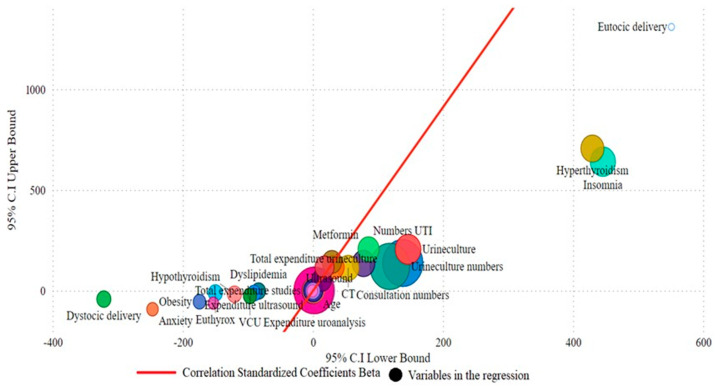
Multiple regression association between expenditure and variables.

Negative correlations were observed between the total expenditure and factors such as obesity (*p* = 0.00020), anxiety (*p* = 0.00021), and dystocic delivery (*p* = 0.011) (Supplementary Table S1). Conversely, positive correlations were noted with variables including age (*p* = 0.001), ASA III scale (*p* = 0.001), and the number of UTIs (*p* = 0.00016). Expenditure increased with the increased use of diagnostic tests, such as ultrasounds (*p* = 0.0009) and CT scans (*p* = 0.0003) (Figure 2).

### 3.7. Multivariate Analysis: Multiple Regression in GA

Total expenditure was negatively correlated with obesity (*p* = 0.001) and positively correlated with ASA III scores (*p* = 0.001), number of UTIs (*p* = 0.002), and use of diagnostic imaging (*p* < 0.05) (Supplementary Table S2 and Figure S1).

### 3.8. Multivariate Analysis: Multiple Regression in GV

Expenditure was negatively correlated with benzodiazepine use (*p* = 0.002) and positively correlated with UTIs (*p* = 0.0021), diabetes (*p* = 0.004), and diagnostic imaging (*p* < 0.05) (Supplementary Table S3 and Figure S2).

### 3.9. Multivariate Analysis: Multiple Regression in GO

Expenditure was inversely correlated with anxiety (*p* = 0.00029) and hypothyroidism (*p* = 0.0004), while it increased with age (*p* = 0.0461) and use of imaging tests (*p* < 0.05) (Supplementary Table S4 and Figure S3).

## 4. Discussion

The allocation of healthcare expenditures remains a contentious topic. According to the World Health Organization, the cost-effectiveness of healthcare interventions can be categorized using the Gross Domestic Product (GDP) per capita. Specifically, an intervention is deemed very cost-effective if its cost is below the GDP per capita, cost-effective if it falls between one and three times the GDP per capita, and not cost-effective if it exceeds three times the GDP per capita [12]. Although Spain lacks an official threshold for cost-effectiveness, estimates commonly range from EUR 30,000 to EUR 45,000 per quality-adjusted life year (QALY) [13].

Among the most clinically significant findings, immunoprophylaxis emerges as a transformative strategy for the management of recurrent urinary tract infections (RUTIs). The MV140 autovaccine and MV140 polybacterial vaccine demonstrated superior cost-effectiveness compared to continuous antibiotic prophylaxis. Notably, the polybacterial vaccine group achieved the lowest recurrence rates (74.6% with 0–1 UTIs) and health costs (EUR 23,629.19) [8]. Similarly, sublingual bacterial vaccination with Uromune^®^ yielded substantial reductions in healthcare resource utilization, including a 71.8% decrease in emergency visits and a 90% reduction in diagnostic tests, culminating in annual cost savings from EUR 1001.1 to EUR 497.1 per patient [7]. These outcomes underscore the potential of immunoprophylaxis as a cost-effective alternative to antibiotics, offering both clinical efficacy and economic benefits [10].

Antibiotic prophylaxis remains a cornerstone of RUTI management. Cost-effectiveness analyses indicate that antibiotics achieve economic viability under specific conditions, such as low drug costs, high recurrence rates in untreated populations, or high antibiotic efficacy [14]. Palmer et al. (2018) emphasized these dynamics, demonstrating the nuanced conditions under which antimicrobial prophylaxis becomes a viable strategy [15]. Despite its established role, antibiotic prophylaxis faces challenges such as increasing antimicrobial resistance and adverse effects, which further highlight the need for alternative approaches [3]. We should remark the value of exploring non-antibiotic strategies, such as estrogen therapy or bacterial vaccines, to minimize antibiotic resistance and enhance quality-adjusted life years (QALYs), ultimately aiding in individualized patient care decisions.

Gyneco-obstetric history significantly influences RUTI-related expenditures. Our analysis corroborates the previous findings that a greater number of eutocic deliveries correlates with increased healthcare costs [16]. Furthermore, advanced age, poor health status (ASA III), and higher diagnostic and therapeutic demands—including urine cultures, imaging studies, and other diagnostic procedures—have emerged as key cost drivers. These factors underscore the complexity of managing RUTIs in diverse patient populations, necessitating individualized approaches to care.

Beyond antibiotics, alternative strategies have garnered attention for their cost-effectiveness and reduced reliance on antimicrobial agents. Methenamine hippurate, for instance, has proven to be a cost-effective alternative, offering slight cost savings and reduced antibiotic resistance risks [17]. Combining immunoprophylaxis with estrogen therapy, particularly in postmenopausal women, further enhances cost-effectiveness by addressing patient-specific needs [14,18]. Additionally, intravesical hyaluronic acid/chondroitin sulfate therapies—although less explored—demonstrate favorable cost-effectiveness profiles when compared to alternatives like DMSO, with our study highlighting the importance of targeted diagnostic approaches such as intravenous urography (IVU) to optimize patient management [19,20,21].

Recent advances in microbiome research have reshaped our understanding of RUTI pathophysiology. Emerging evidence highlights the interplay between gut dysbiosis and the urinary microbiome, suggesting that interventions targeting gut health, such as probiotics or immunotherapies, could complement traditional RUTI prevention strategies [22]. These findings may offer a holistic framework for managing infections by addressing underlying microbial imbalances.

In our study, sublingual immunoprophylaxis (Group V) demonstrated superior economic and clinical outcomes compared to the other strategies. With a mean expenditure of EUR 151.37 for successive visits and an average of just 1.09 visits per patient, this approach significantly reduced healthcare resource utilization while maintaining efficacy. These findings highlight the transformative potential of immunoprophylaxis as a sustainable and cost-effective alternative to traditional antibiotic prophylaxis, especially given the ongoing challenges of antibiotic resistance.

The analysis also aimed to identify cost differences between prophylactic protocols, a key objective in this study. The comparison of Groups A, V, and O reveals significant variability in both expenditures and patient outcomes. For instance, Group A (antibiotic prophylaxis) demonstrated that ciprofloxacin (GAa) was the most cost-effective antibiotic regimen, with the lowest per-dose costs (EUR 2.31) and fewer successive visits (mean: 3.45), whereas amoxicillin (GAe) incurred higher costs (EUR 8.73 per dose). On the other hand, Group O (non-antibiotic measures) exhibited a wide range of cost-effectiveness, with vaginal estrogens (GOd) reducing the number of successive visits (mean: 3.71) at a relatively lower per-dose expenditure (EUR 421.50).

These cost comparisons underline the economic advantage of non-antibiotic strategies and immunoprophylaxis in specific patient subgroups. For instance, Uromune^®^ (Group V) emerged as a highly efficient option, with significantly lower healthcare costs and a marked reduction in successive visits. Such findings emphasize the need for tailored prophylactic strategies that align with patient characteristics and healthcare system priorities.

This study has several limitations that should be acknowledged. First, postcoital prophylaxis was not included in the analysis, as it is not a routine practice in our healthcare center. While postcoital prophylaxis is recognized as a viable strategy for preventing recurrent urinary tract infections (RUTIs) in some clinical settings [23], its exclusion limits the generalizability of our findings to centers or populations where this approach is commonly used. Additionally, certain aspects of the study may introduce potential biases, such as no uniform protocol in imaging studies. Future research should aim to include a broader range of prophylactic approaches, including postcoital prophylaxis, and involve multicenter collaborations to enhance the external validity of the findings.

## 5. Conclusions

The total costs associated with RUTIs are significantly influenced by age, poorer health status, and the use of diagnostic tests such as ultrasounds and CT scans. Among the prophylactic strategies, sublingual immunoprophylaxis demonstrated the highest cost-effectiveness by significantly reducing emergency visits, follow-up appointments, infection recurrence, and diagnostic test needs. While non-antibiotic adjuvant measures, such as intravesical hyaluronic acid instillations, show promise in selected patients, immunoprophylaxis remains the most efficient strategy overall. Personalized protocols prioritizing immunoprophylaxis can optimize healthcare resource utilization and improve outcomes in RUTI management.

## Figures and Tables

**Table 1:** Age, body mass index (BMI), and duration of RUTI clinical evolution period in different groups and subgroups of prophylaxis. ANOVA test was employed in statistical analysis.

Group	n	%	Age (Years)					BMI (kg/m^2^)					Years Suffering RUTI			
			Mean	Sd	Median	Range	*p*	Mean	Sd	Median	Range	*p*	Mean	Sd	Median	Range
GA	444	27.51	57.25	20.65	63	18–87	0.0005	27.97	4.43	26.67	19.53–39.45	0.0002	5.4	4	5.4	5–5.4
GV	732	45.35	52.27	18.43	59	18–93		27.49	3.59	26.78	19.33–38.55		4.4	6.00	4.4	4–4.4
GO	438	27.14	60.57	16.65	62	19–88		28.43	3.73	28.14	21.83–39.65		5.7	1	5.7	5–5.7

## Data Availability

The original contributions presented in this study are included in the article/Supplementary Materials. Further inquiries can be directed to the corresponding author.

## Supplementary Materials

The following supporting information can be downloaded at: https://www.mdpi.com/article/10.3390/microorganisms13020393/s1, Figure S1: Multiple regression, association between expenditure and variables in GA; Figure S2: Multiple regression, association between expenditure and variables in GV; Figure S3: Multiple regression, association between expenditure and variables in GO; Table S1: Multivariate analysis: Multiple regression: association between expenditure and the variables; Table S2: Multivariate analysis: Multiple regression: association between expenditure and the variables in GA; Table S3: Multivariate analysis: Multiple regression: association between expenditure and the variables in GV; Table S4: Multivariate analysis: Multiple regression: association between total expenditure and the variables in GO.

## References

[1] Patel D. (2024). Intravesical Therapies for Recurrent Urinary Tract Infections: A Systematic Review. Cureus.

[2] Nickel C. (2005). Management of Urinary Tract Infections: Historical Perspective and Current strategies: Part I—Before antiobiotics. J. Urol..

[3] Bonkat G., Bartoletti R., Bruyère F., Cai T., Geerlings S.E., Köves B., Kranz J., Schubert S., Pilatz A., Veeratterapillay R. (2024). EAU Guidelines on Urological Infections. European Associaion of Urology Guidelines.

[4] Keating K.N., Perfetto E.M., Subedi P. (2005). Economic burden of uncomplicated urinary tract infections: Direct, indirect and intangible costs. Expert Rev. Pharmacoeconomics Outcomes Res..

[5] Lorenzo-Gómez M.F., Foley S., Nickel J.C., García-Cenador M.B., Padilla-Fernández B.Y., González-Casado I., Martínez-Huélamo M., Yang B., Blick C., Ferreira F. (2022). Sublingual MV140 for Prevention of Recurrent Urinary Tract Infections. NEJM Evid..

[6] Lorenzo-Gómez M.F., Padilla-Fernández B., García-Criado F.J., Mirón-Canelo J.A., Gil-Vicente A., Nieto-Huertos A., Silva-Abuin J.M. (2013). Evaluation of a therapeutic vaccine for the prevention of recurrent urinary tract infections versus prophylactic treatment with antibiotics. Int. Urogynecol. J..

[7] Carrión-López P., Martínez-Ruiz J., Giménez-Bachs J.M., Fernández-Anguita P.J., Díaz de Mera-Sánchez Migallón I., Legido-Gómez O., Rico-Marco S., Lorenzo-Sánchez M.V., Salinas-Sánchez A.S. (2022). Cost-Effectiveness of a Sublingual Bacterial Vaccine for the Prophylaxis of Recurrent Urinary Tract Infections. Urol. Int..

[8] Ramírez-Sevilla C., Gómez-Lanza E., Llopis-Manzanera J., Cetina-Herrando A., Puyol-Pallàs J.M. (2023). Effectiveness and health cost analysis between immunoprophylaxis with MV140 autovaccine, MV140 vaccine and continuous treatment with antibiotics to prevent recurrent urinary tract infections. Actas Urol. Esp..

[9] Zilberberg M., Shorr A. (2010). Understanding cost-effectiveness. Clin. Microbiol. Infect..

[10] Gaitonde S., Malik R.D., Zimmern P.E. (2019). Financial Burden of Recurrent Urinary Tract Infections in Women: A Time-driven Activity-based Cost Analysis. Urology.

[11] NCSS NCSS Statistical Software.

[12] Fraga-Fuentes M., López-Sánchez P., Andrés-Navarro M., Valenzuela-Gómez J., Perez-Fernández E., Heredia-Benito M. (2014). Evaluación económica de medicamentos: Puntos a considerar para no perderse. Bol. Farmacoter. Castilla Mancha.

[13] Vallejo-Torres L., García-Lorenzo B., Castilla I., Valcárcel-Nazco C., García-Pérez L., Linertová R., Serrano-Aguilar P., de Tenerife S.C. (2015). Valor Monetario de un Año de Vida Ajustado por Calidad: Estimación empírica del coste de oportunidad en el Sistema Nacional de Salud. Ministerio de Sanidad, Servicios Sociales e Igualdad. Servicio de Evaluación del Servicio Canario de la Salud; 2015. Informes de Evaluación de Tecnologías Sanitarias. Ministerio de Sanidad SSeISCdlS.

[14] Eells S.J., Bharadwa K., McKinnell J.A., Miller L.G. (2013). Recurrent urinary tract infections among women: Comparative effectiveness of 5 prevention and management strategies using a Markov chain Monte Carlo model. Clin. Infect. Dis..

[15] Palmer L.S., Seideman C.A., Lotan Y. (2018). Cost-effectiveness of antimicrobial prophylaxis for children in the RIVUR trial. World J. Urol..

[16] Vanaclocha-Ferrer C., Padilla-Fernandez B.Y., Marquez-Sanchez M.T., Garcia-Sanchez M.H., Rodriguez-Martin M.D., Hernandez-Navarro N., Domenech-Perez C., Valverde-Martinez L.-S., Flores-Fraile M.-C., Huélamo M.M. (2021). Relationship between obstetric history and recurrent urinary infections. Sci. Rep..

[17] Harding C., Chadwick T., Homer T., Lecouturier J., Mossop H., Carnell S., King W., Abouhajar A., Vale L., Watson G. (2022). Methenamine hippurate compared with antibiotic prophylaxis to prevent recurrent urinary tract infections in women: The ALTAR non-inferiority RCT. Health Technol. Assess..

[18] Fox K.A., Lokken E.M., Reed S.D., Rahn D.D. (2021). Evaluation of systemic estrogen for preventing urinary tract infections in postmenopausal women. Menopause.

[19] Goddard J.C., Janssen D.A. (2018). Intravesical hyaluronic acid and chondroitin sulfate for recurrent urinary tract infections: Systematic review and meta-analysis. Int. Urogynecol. J..

[20] Boronat Catalá J., García Tello A., González Montes L., Ruiz Graña S., Torres Pérez D., Llanes González L. (2021). Efectividad y seguridad del ácido hialurónico intravesical para el control de los síntomas en cistopatías crónicas [Effectiveness and safety of intravesical hyaluronic acid for symptom control in chronic bladder diseases]. Arch. Esp. Urol..

[21] Cervigni M., Sommariva M., Tenaglia R., Porru D., Ostardo E., Giammò A., Trevisan S., Frangione V., Ciani O., Tarricone R. (2017). A randomized, open-label, multicenter study of the efficacy and safety of intravesical hyaluronic acid and chondroitin sulfate versus dimethyl sulfoxide in women with bladder pain syndrome/interstitial cystitis. Neurourol. Urodyn..

[22] Brigida M., Saviano A., Petruzziello C., Manetti L.L., Migneco A., Ojetti V. (2024). Gut Microbiome Implication and Modulation in the Management of Recurrent Urinary Tract Infection. Pathogens.

[23] Houston C.G., Azar W.S., Huang S.S., Rubin R., Dorris C.S., Sussman R.D. (2024). A Cost Savings Analysis of Topical Estrogen Therapy in Urinary Tract Infection Prevention Among Postmenopausal Women. Urol. Pract..

