# Supplementary material for: Evaluation and Analysis of Costs Associated with Prophylaxis of Recurrent Urinary Tract Infections (RUTIs) in Women
Source: Microorganisms. 2025 Feb 11;13(2):393. doi: 10.3390/microorganisms13020393 (PMC11857891; doi:10.3390/microorganisms13020393)
Supplement: Supplementary file 1 [file microorganisms-13-00393-s001.zip › microorganisms-3390359-supplementary.pdf]

Supplementary tables.

Supplementary Table S1.- Multivariate analysis: Multiple regression: association between expenditure and the variables.

Variables	Unstandardized Coefficients Beta	Standardized Coefficients Beta	Sig.	95% I.C Lower Bound	95% I.C Upper Bound
Age	1.334	0.086	0.001	0.551	2.116
ASA	33.966	0.081	0.001	13.449	54.482
Visits (n)	137.990	1.000	0.0002	137.990	137.990
UTIs (n)	137.990	1.000	0.00016	137.990	137.990
Urinalysis expenditure	2.323	1.000	0.0035	2.323	2.323
Urine culture	119.559	0.967	0.0004	118.032	121.086
Urine cytology	86.246	0.074	0.003	29.123	143.368
Diabetes	67.788	0.070	0.008	17.882	117.694
Dyslipidemia	-42.383	-0.056	0.044	-83.677	-1.088
Obesity	-114.208	-0.090	0.00020	-174.420	-53.996
Anxiety	-168.977	-0.106	0.00021	-246.640	-91.314
Insomnia	543.338	0.263	0.0009	445.631	641.045
Hypothyroidism	-68.655	-0.063	0.010	-120.643	-16.668
Hyperthyroidism	567.886	0.198	0.0001	429.551	706.221
Eutocic delivery	930.147		0.0002	551.004	1309.290
Dystocic delivery	-181.217	-0.063	0.011	-321.459	-40.975
Abdominal surgery	-50.855	-0.067	0.012	-90.429	-11.282
Metformin	146.257	0.131	0.0003	85.457	207.058
Euthyrox	-80.268	-0.059	0.024	-150.061	-10.475

Benzodiazepines	-107.026	-0.129	0.0006	-153.122	-60.929
Statin	74.776	0.100	0.001	32.454	117.099
Ultrasound	106.853	0.175	0.0009	77.704	136.001
CT (n)	83.956	0.136	0.0003	54.185	113.726
VCU (n)	-61.194	-0.082	0.001	-96.863	-25.526

ASA: American Society of Anesthesiologists Physical Status Classification Scale (32). UTI: urinary tract infection. CT: Computerized tomography. VCU: voiding cystourethrography. C.I.: confidence interval.

Supplementary Table S2.- Multivariate analysis: Multiple regression: association between expenditure and the variables in GA.

Variables	Unstandardized Coefficients Beta	Standardized Coefficients Beta	Sig.	95% I.C Lower Bound	95% I.C Upper Bound
ASA	75.435	0.164	0.001	33.135	117.734
Urinalysis expenditure	2.323	1.000	0.0001	2.323	2.323
UTIs (n)	137.990	1.000	0.002	137.990	137.990
Urine culture	120.973	0.970	0.0002	118.133	123.814
Obesity	-178.982	-0.157	0.001	-279.872	-78.092
Insomnia	694.060	0.407	0.0001	554.647	833.473
Hyperthyroidism	517.687	0.273	0.0008	344.360	691.015
Dystocic delivery	-338.244	-0.137	0.004	-567.972	-108.516
Abdominal surgery	-94.855	-0.127	0.013	-169.508	-20.202

ACEi	-124.487	-0.132	0.045	-245.930	-3.044
Euthyrox	-234.267	-0.182	0.001	-367.413	-101.121
Benzodiazepines	-126.324	-0.148	0.013	-226.082	-26.567
Ultrasound	70.307	0.113	0.015	13.611	127.002
CT	150.974	0.235	0.0008	91.604	210.345
VCU (n)	-135.055	-0.159	0.001	-213.987	-56.124

ASA: American Society of Anesthesiologists Physical Status Classification Scale (32). UTI: urinary tract infection. CT: computerized tomography. VCU: voiding cystourethrography. C.I.: confidence interval. ACEi: angiotensin converting enzyme inhibitor.

Supplementary Table S3.- Multivariate analysis: Multiple regression: association between expenditure and the variables in GV.

Variables	Unstandardized Coefficients Beta	Standardized Coefficients Beta	Sig.	95% I.C Lower Bound	95% I.C Upper Bound
Visits (n)	137.990	1.000	0.0001	137.990	137.990
UTI (n)	137.990	1.000	0.0021	137.990	137.990
Diabetes	134.120	0.122	0.004	43.703	224.537
Hyperthyroidism	585.003	0.148	0.0009	292.144	877.861
Metformin	220.185	0.180	0.00014	121.439	318.930
Benzodiazepines	-109.040	-0.130	0.002	-178.336	-39.745

Statin	81.436	0.102	0.025	10.321	152.551
Ultrasound	58.130	0.099	0.007	15.777	100.483
VCU	-54.050	-0.080	0.031	-103.214	-4.886

UTI: urinary tract infection. VCU: voiding cystourethrography. C.I.: confidence interval.

Supplementary Table S4.- Multivariate analysis: Multiple regression: association between total expenditure and the variables in GO.

Variables	Unstandardized Coefficients Beta	Standardized Coefficients Beta	Sig.	95% I.C Lower Bound	95% I.C Upper Bound
Age	1,704	0,102	0,046	0,032	3,377
Anxiety	-289,979	-0,180	0,00029	-446,184	-133,775
Insomnia	623,608	0,306	0,0003	433,615	813,602
Hypothyroidism	-165,699	-0,173	0,0004	-257,909	-73,489
ACEi	-123,505	-0,114	0,025	-231,390	-15,620
Diuretic	-286,999	-0,148	0,006	-491,101	-82,896
Metformin	108,422	0,112	0,049	0,681	216,163
Benzodiazepines	-93,349	-0,117	0,027	-175,900	-10,799
Statin	101,662	0,139	0,008	27,036	176,288
Analgesic	-152,736	-0,126	0,019	-280,279	-25,194

PPI	102,323	0,094	0,079	-11,927	216,572
Ultrasound	278,493	0,409	0,0001	219,244	337,742
CT	90,231	0,146	0,001	36,742	143,721
Urography	-321,347	-0,096	0,031	-612,456	-30,238

ASA: American Society of Anesthesiologists Physical Status Classification Scale. UTI: urinary tract infection. CT: computerized tomography. VCU: voiding cystourethrography. C.I.: confidence interval. ACEi: angiotensin converting enzyme inhibitor. PPI: Proton-pump inhibitor.

Supplementary figures.

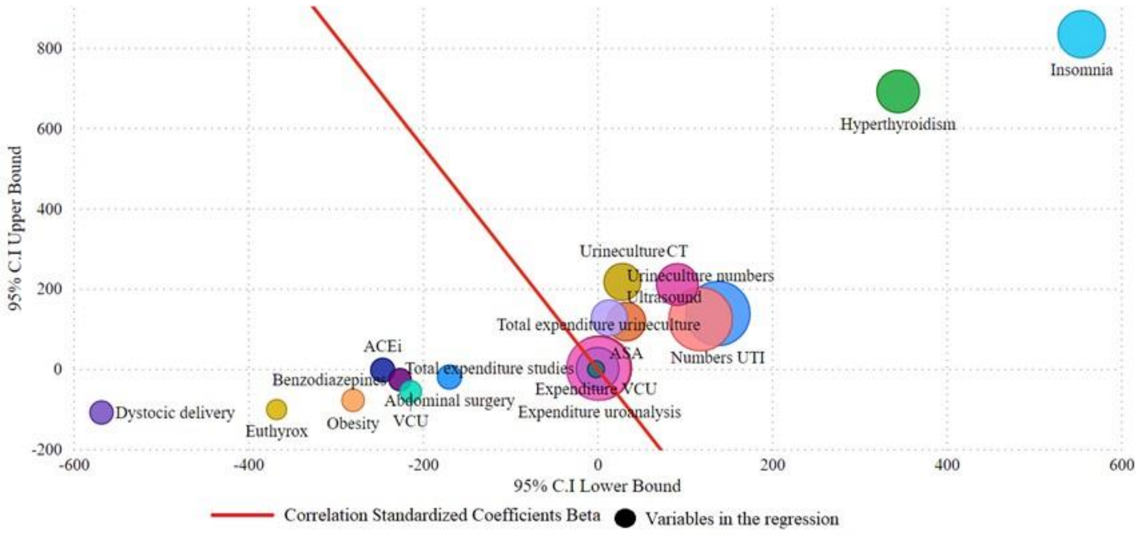

Figure S1: Multiple regression, association between expenditure and variables in GA.

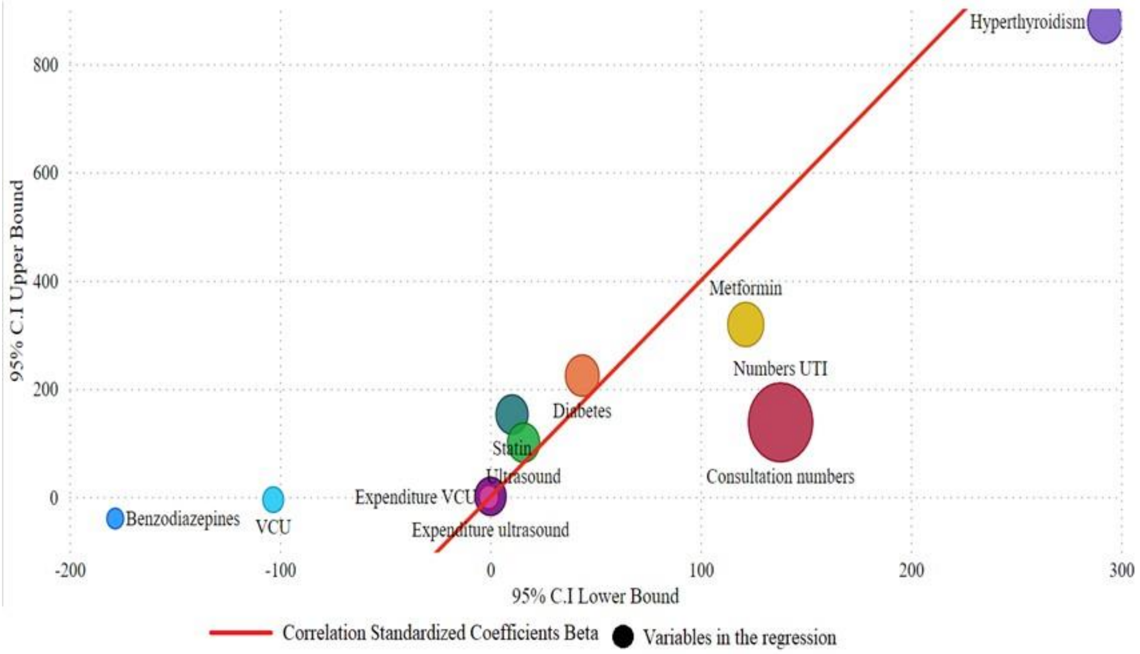

Figure S2: Multiple regression, association between expenditure and variables in GV.

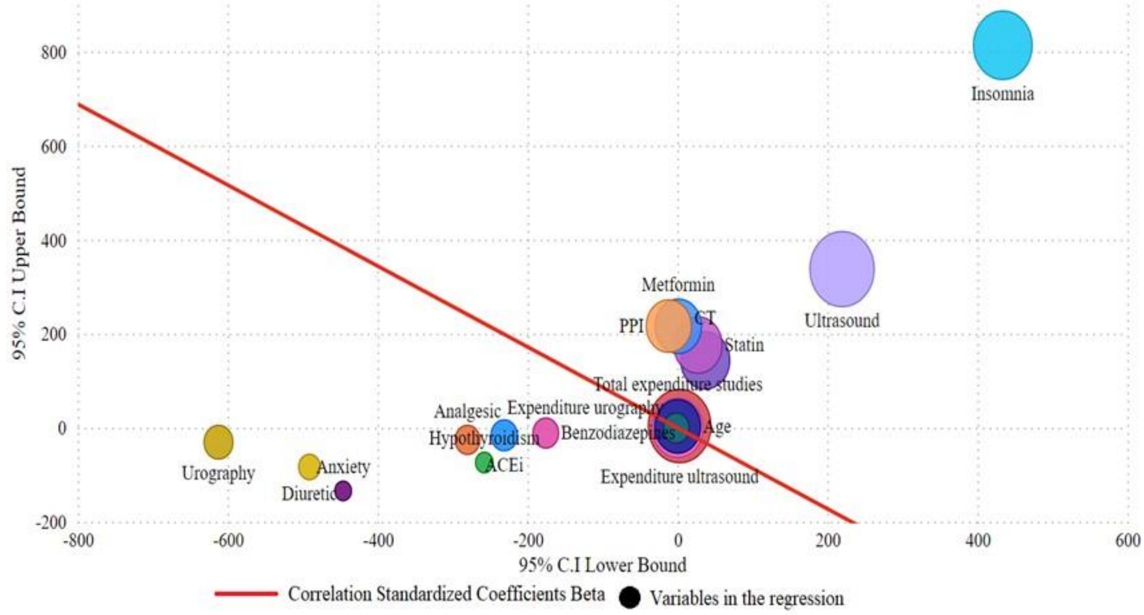

Figure S3: Multiple regression, association between expenditure and variables in GO.
